# Sex differences in VTA GABA transmission and plasticity during opioid withdrawal

**DOI:** 10.1038/s41598-023-35673-9

**Published:** 2023-05-25

**Authors:** Daniel J. Kalamarides, Aditi Singh, Shannon L. Wolfman, John A. Dani

**Affiliations:** Department of Neuroscience, Perelman School of Medicine, Mahoney Institute for Neurosciences, University of Pennsylvania, 415 Curie Blvd, Philadelphia, PA 19104 USA

**Keywords:** Long-term potentiation, Neurotransmitters, Inhibition, Neurophysiology, Addiction

## Abstract

The effectiveness of current treatments for opioid use disorder (OUD) varies by sex. Our understanding of the neurobiological mechanisms mediating negative states during withdrawal is lacking, particularly with regard to sex differences. Based on preclinical research in male subjects, opioid withdrawal is accompanied by increased gamma-aminobutyric acid (GABA) release probability at synapses onto dopamine neurons in the ventral tegmental area (VTA). It is unclear, however, if the physiological consequences of morphine that were originally elucidated in male rodents extend to females. The effects of morphine on the induction of future synaptic plasticity are also unknown. Here, we show that inhibitory synaptic long-term potentiation (LTP_GABA_) is occluded in the VTA in male mice after repeated morphine injections and 1 day of withdrawal, while morphine-treated female mice maintain the ability to evoke LTP_GABA_ and have basal GABA activity similar to controls. Our observation of this physiological difference between male and female mice connects previous reports of sex differences in areas upstream and downstream of the GABA-dopamine synapse in the VTA during opioid withdrawal. The sex differences highlight the mechanistic distinctions between males and females that can be targeted when designing and implementing treatments for OUD.

## Introduction

The prevalence and impact of opioid misuse have ballooned over the past decades, leading to 1.1 million people receiving treatment for opioid use disorder (OUD) in 2020^1^. Current therapeutic strategies for OUD are limited by high susceptibility to relapse^2–4^ and individual response differences^3,5^. One such individual difference is that men and women differ in behavioral and physiological aspects of OUD, including the rate of progression from initial drug use to treatment initiation and withdrawal symptoms^6^. In turn, these differences likely underlie or magnify sex and gender differences in treatment efficacy^7,8^. Consequently, additional treatment approaches that target individual and sex differences during opioid withdrawal are in high demand^9^. Despite a 2016 policy from the National Institutes of Health that called for addressing sex as a biological variable in research design, analysis, and reporting, many topics have continued to go understudied^10^.

The ventral tegmental area (VTA) contains dopamine neurons that have long been implicated in addiction-related behavior^11^. Dopaminergic activity is increased during acute opioid exposure^11^ and decreased during withdrawal^12,13^. Since these discoveries, it has been shown that gamma-aminobutyric acid (GABA) transmission in the VTA mediates the acute effects of opioids on dopamine neurons^14–16^. During opioid withdrawal, there is a rebound of the cyclic adenosine monophosphate (cAMP) signaling pathway controlled by mu-opioid receptors, leading to increased GABA release in the VTA in male rodents^17,18^. Increasing VTA GABA release via optogenetic activation of GABA neurons is sufficient to drive aversion in drug naïve animals^19,20^, indicating GABA release in the VTA may be central to negative behavioral states during opioid withdrawal. Beyond changes in baseline GABA activity, studies have also demonstrated that a single morphine injection temporarily impairs long-term potentiation at GABA synapses (LTP_GABA_) in the VTA in male rodents^21^. Inhibitory plasticity such as LTP_GABA_ has been understudied compared to excitatory LTP, but it has direct impact on subsequent inhibitory control over dopaminergic firing patterns^22^. Despite the critical role of inhibitory signaling in the VTA in addiction-related behavior, very few studies have investigated VTA GABA activity in females after repeated opioids. In contrast, brain areas upstream of the VTA known to contribute to reward and aversion, including the bed nucleus of the stria terminalis (BNST)^23^ and the rostromedial tegmental nucleus (RMTg)^24,25^, are sexually dimorphic during opioid withdrawal^26,27^ and make GABAergic projections to the VTA^28^. While there are not major intrinsic physiological sex differences in VTA dopamine neurons^29^, potential sex differences after opioids remain a knowledge gap that could be a crucial stepping stone for developing effective OUD treatment^30,31^.

In this paper, we evaluated sex as a biological variable in reward circuitry during opioid withdrawal. Given that the VTA receives inhibitory inputs from several sexually dimorphic brain regions and is key to reward and aversion-related behavior, we hypothesized that GABA inputs to VTA dopamine neurons would differ between males and females during withdrawal. To address this question, male and female mice were subjected to a 5-day morphine injection paradigm. Next, they were tested for multiple measures of VTA GABA transmission after 1 day of withdrawal. In male mice, but not females, withdrawal increased GABA release probability in the VTA based on a decrease in paired pulse ratio (PPR) and an increase in spontaneous inhibitory post-synaptic current frequency (sIPSC). Furthermore, morphine treatment led to impairment of a form of inhibitory plasticity in males, while inhibitory plasticity remained intact in females. Together, the results indicate that repeated opioid administration and subsequent withdrawal caused diverging effects in VTA physiology for males and females, which may contribute to sex differences in behaviors related to OUD.

## Results

### VTA GABA release probability increases in male mice during morphine withdrawal but does not increase in female mice

**Figure 1:**
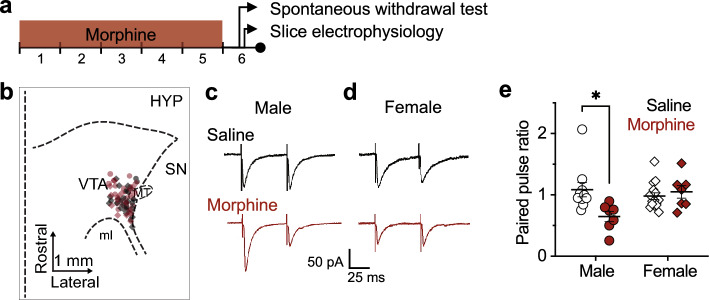
Morphine withdrawal decreases the GABAergic paired pulse ratio (PPR) in male VTA dopamine neurons but not in females. (**a**) Timeline of experiments. Male and female adult C57 mice were exposed to morphine via i.p. injection (10 mg/kg) twice daily for 5 days. 18–24 h after the final injection, mice were observed for spontaneous withdrawal signs and then brain slices containing the ventral tegmental area (VTA) were cut for electrophysiology experiments. (**b**) Didactic horizontal slice view of recording locations of cells in the lateral VTA from male (circles) and female (diamonds) mice treated with morphine (red) or saline (gray). *HYP* hypothalamus, *SN* substantia nigra, *ml* medial lemniscus, *MT* medial terminal nucleus of accessory optic tract. Dopamine neurons were voltage clamped at − 60 mV throughout the experiments unless otherwise noted. GABA activity was isolated by bathing slices with aCSF containing DNQX (10 μM) and strychnine (1 μM) to block AMPA and glycine activity. (**c**) Example traces containing paired electrical stimulation of GABA afferents in cells from male and (**d**) female mice. (**e**) PPRs (amplitude of evoked current from pulse 2/pulse 1) in dopamine neurons from male and female mice treated with morphine or saline. There was a significant interaction between sex and drug treatment. Cells from morphine-treated male mice (n = 8 cells, 7 mice) had a decreased paired pulse ratio (PPR) relative to cells from saline controls (n = 13 cells, 10 mice) while cells from morphine-treated females (n = 8 cells, 7 mice) did not differ from saline controls (n = 19 cells, 15 mice). *p < 0.05.

Prior studies have shown that withdrawal from opioids increases GABA release probability in the VTA in male rodents^17^, as evidenced by an increase in the frequency of spontaneous IPSCs and a decrease in PPR^17,32^. However, the ways in which VTA GABA transmission and plasticity in females are affected by opioid withdrawal are unknown. To address this gap, we used brain slice electrophysiology to record from lateral VTA dopamine neurons in both male and female mice after they received twice daily morphine injections (10 mg/kg, intraperitoneal) for 5 days followed by 18 h withdrawal (Fig. 1a,b). During recordings, paired electrical stimulations separated by 50 ms were applied to GABA afferents while dopamine neurons were clamped at − 60 mV, and the resulting IPSC amplitudes were analyzed for males (Fig. 1c) and females (Fig. 1d). Morphine treatment led to a 39% decrease in PPR compared to saline controls in males, but morphine treatment did not alter PPR in females (2-way ANOVA: sex F(1, 35) = 2.62, *p* = 0.11, drug treatment F(1, 35) = 4.08, *p* = 0.05, sex × drug treatment interaction F(1, 35) = 7.79, *p* < 0.01; Tukey post hoc test for multiple comparisons: male *p* = 0.01, female *p* = 0.94; Fig. 1e). These findings indicate that the known increase in presynaptic GABA release probability seen in male mice during opioid withdrawal does not generalize to female mice under these conditions.

**Figure 2:**
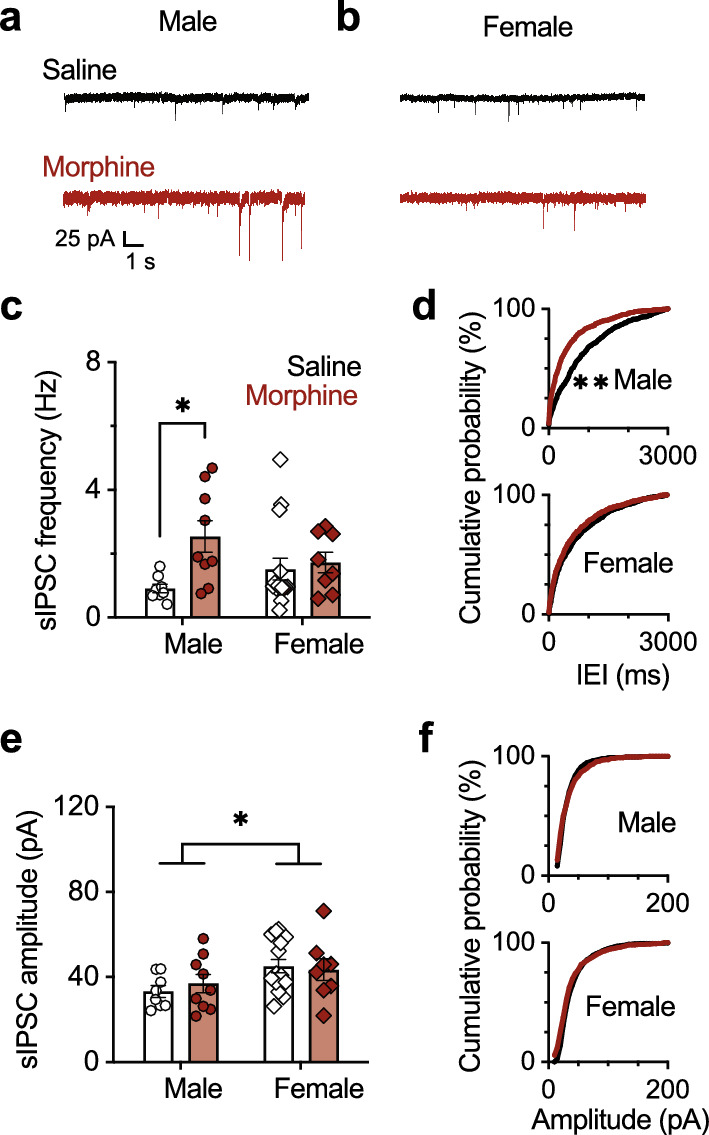
Morphine withdrawal sex-dependently impacts VTA GABA spontaneous inhibitory postsynaptic activity onto VTA dopamine neurons. (**a**) Representative traces containing spontaneous inhibitory postsynaptic currents (sIPSC) for males and (**b**) females. (**c**) Summary sIPSC frequency data for males and females treated with morphine or saline. Morphine-treated males (n = 14 cells, 9 mice) had increased sIPSC frequency relative to saline-treated males (n = 10 cells, 8 mice), and there was not a significant effect of morphine treatment on female cells (n = 15 cells, 8 mice) relative to saline treatment (n = 21 cells, 15 mice). (**d**) Cumulative probability histogram of sIPSC inter-event intervals for cells from male (top) and (bottom) female mice. Morphine treatment led to an increase in sIPSC frequency based on inter-event frequency distribution analysis in males but did not alter inter-event frequency distribution in females. (**e**) Summary sIPSC amplitude data for males and females treated with morphine or saline. Cells from females had higher sIPSC amplitudes than males. However, there was not a main effect of drug treatment or an interaction. (**f**) Cumulative probability histogram of sIPSC amplitude for cells from male (top) and female (bottom) mice. There was not a different in sIPSC amplitude based on distribution analysis in males or females. *p < 0.05, **p < 0.01.

To confirm this sex difference, we measured spontaneous IPSCs onto VTA dopamine neurons in males (Fig. 2a) and females (Fig. 2b). As predicted by our PPR results, morphine-treated male mice averaged greater than a 100% increase in sIPSC frequency compared to saline controls and there was not a significant difference between morphine and saline-treated female mice (2-way ANOVA: sex F(1, 36) = 0.08, *p* = 0.78, drug treatment F(1, 36) = 5.80, *p* = 0.02, sex × drug treatment interaction F(1, 36) = 3.44, *p* = 0.07; Tukey post hoc test for multiple comparisons: male *p* = 0.04, female *p* = 0.99; Fig. 2c). Consistent with the analysis of average frequencies, morphine withdrawal decreased sIPSC inter-event interval cumulative probability in males but not females (K-S test: male *p* < 0.01, female *p* = 0.80; Fig. 2d).

Spontaneous IPSC amplitudes from the same recordings were also analyzed. Cells from female mice averaged 10 pA larger currents than males, however, morphine did not alter sIPSC amplitude (2-way ANOVA: sex F(1, 36) = 5.45, *p* = 0.03, drug treatment F(1, 36) = 0.07, *p* = 0.79, sex × drug treatment interaction F(1, 36) = 0.47, *p* = 0.49; Tukey post hoc test for multiple comparisons: male *p* = 0.99, female *p* = 0.99; Fig. 2e). Morphine withdrawal did not affect sIPSC amplitude cumulative probability in males or females (K-S test: male *p* = 0.12, female *p* = 0.35; Fig. 2f). The sIPSC results indicate that morphine withdrawal leads to an increased frequency in males, but not females, and that amplitude is unaffected by morphine treatment. Together, these PPR and sIPSC results indicate that VTA GABA circuitry is differentially impacted by opioid withdrawal in males and females and that this difference is likely mediated by presynaptic mechanisms.

### Inhibitory plasticity (LTP_GABA_) in the VTA after saline or morphine

**Figure 3:**
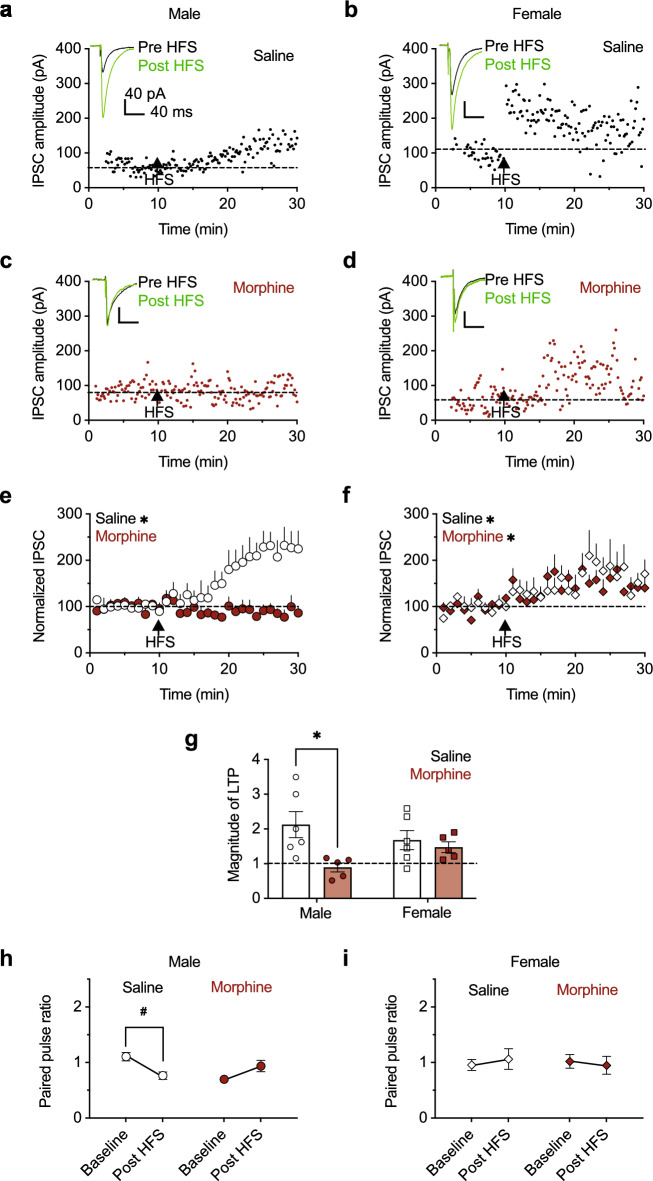
Morphine impairs inhibitory plasticity (LTP_GABA_) in males but not females. (**a**) Example of a single recording of evoked IPSCs during a 10-min pre high frequency stimulation (HFS) baseline and a 20-min post HFS recording in a cell from a saline-treated male, (**b**) saline-treated female, (**c**) morphine-treated male, and (**d**) morphine-treated female. Dashed horizontal lines refer to the cell’s average IPSC amplitude during baseline. Inset: representative averaged trace before and after HFS. (**e**) Average normalized IPSCs during baseline (open circles) and post HFS (filled red circles) in males. IPSCs are potentiated after high frequency stimulation in cells from saline-treated males (n = 8 cells, 6 mice), but morphine-treated males (n = 5 cells, 5 mice) do not show potentiation. (**f**) Average normalized IPSCs during baseline (open diamonds) and post HFS (filled red diamonds) in females. Cells from female mice treated with saline (n = 7 cells, 7 mice) or morphine (n = 5 cells, 5 mice) exhibit LTP. (**g**) Comparison of LTP_GABA_ magnitude during the 5-min period 15–20 min following HFS for all groups. (**h**) Mean PPR during baseline and post HFS in cells from male and (**i**) female mice treated with morphine or saline. HFS decreased PPR in cells from males treated with saline (n = 8 cells, 6 mice), but not with morphine (n = 5 cells, 5 mice). HFS did not decrease PPR in cells from females treated with saline (n = 7 cells, 7 mice) or morphine (n = 5 cells, 5 mice). *p < 0.05, ^#^p = 0.05.

Little is known about how synaptic plasticity in the VTA is altered following repeated opioid exposure and how sex influences these changes^33^. A single injection of morphine is sufficient to impair LTP_GABA_^21^, but it is not clear whether this effect persists after repeated morphine administration or whether it is a temporary signature of the initial opioid exposure. Therefore, we examined LTP_GABA_ in the VTA of male and female mice following 5 days of twice-daily morphine treatment (Fig. 3a–d). Recordings were done in lateral VTA dopamine neurons, and IPSCs were evoked using electrical stimulation. Evoked IPSC amplitudes were measured before and after high frequency stimulation (HFS; two, 1-s, 100 Hz trains separated by 20 s). Consistent with the findings after acute morphine, HFS in cells from saline-treated male mice potentiated IPSCs, while HFS in cells from morphine-treated male mice did not potentiate IPSCs (paired t-test: saline male t(5) = 2.84, *p* = 0.04, morphine male t(4) = 0.66, *p* = 0.54; Fig. 3e), indicating that morphine continued to impair LTP_GABA_ after 1 day of withdrawal. In contrast to results from male mice, IPSCs were significantly potentiated after HFS in cells from both saline and morphine-treated female mice (paired t-test: saline female t(6) = 2.71, *p* = 0.04, morphine female t(5) = 3.05, *p* = 0.03; Fig. 3f). In a direct comparison between groups, morphine-treated males had impaired LTP_GABA_ relative to saline-treated males_,_ while LTP_GABA_ did not differ between saline-treated males, saline-treated females, and morphine treated females (2-way ANOVA: sex F(1, 18) = 0.06, *p* = 0.81, drug treatment F(1, 18) = 6.912, *p* = 0.02, sex × drug treatment interaction F(1, 18) = 3.52, *p* = 0.07; Tukey post hoc test for multiple comparisons: saline male vs morphine male *p* = 0.03, saline male vs saline female *p* = 0.49, saline male vs morphine female *p* = 0.31; Fig. 3g). These results show that the ability to evoke inhibitory plasticity is altered in a sex-dependent manner during morphine withdrawal.

The mechanism by which LTP_GABA_ in the VTA increases presynaptic GABA release is via postsynaptic, NMDA-dependent nitric oxide (NO) synthesis and diffusion to the presynapse, where it then promotes GABA release via cyclic guanosine monophosphate (cGMP)^21^. This presynaptic plasticity mechanism leads to a decrease in PPR following HFS in drug naïve animals^21^. Therefore, we analyzed PPR before and after HFS in both sexes after drug treatment. Consistent with the LTP_GABA_ data, PPR was decreased following HFS in saline-treated male mice, but unchanged following HFS in morphine-treated males (paired t-test: saline male t(4) = 2.85, p = 0.05, morphine male t(4) = 1.45, *p* = 0.22; Fig. 3h). Interestingly, despite intact LTP_GABA_ in saline and morphine-treated females, neither group had a decreased PPR following HFS (paired t-test: saline female t(7) = 0.43, *p* = 0.68; morphine female t(4) = 0.61, *p* = 0.57; Fig. 3i), indicating that females may have distinct mechanisms for LTP_GABA_ induction in VTA dopamine neurons.

### Opioid dependence following morphine injection paradigm

**Figure 4:**
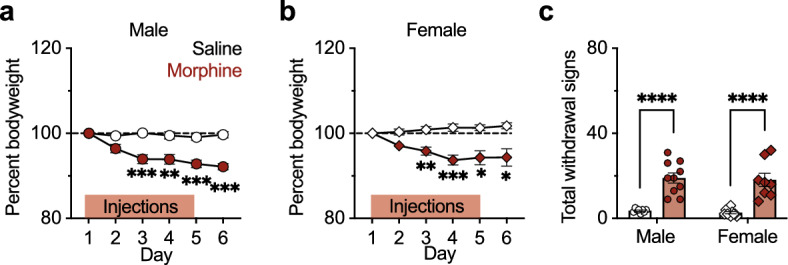
Both male and female mice are opioid dependent following morphine administration. (**a**) Male mouse weight decreased for morphine-treated animals (n = 10) compared to saline controls (n = 12). (**b**) Female mouse weight decreased for morphine-treated animals (n = 8) compared to saline controls (n = 15). (**c**) Total withdrawal signs including wet dog shakes, jumps, abnormal posture, and teeth chattering observed for 20 min, 18–24 h after the final morphine or saline injection. Morphine treatment led to significantly more withdrawal signs for both males (n = 10) and females (n = 8) compared to controls (male saline n = 8; female saline n = 10). *p < 0.05, **p < 0.01, ***p < 0.001, ****p < 0.0001.

Male and female mice have different sensitivities to a morphine dose, even when controlling for weight^34,35^. To verify that the 5-day morphine injection procedure caused opioid dependence and subsequence withdrawal in both sexes, mice were weighed daily and tested for signs of spontaneous withdrawal 18–24 h after the last morphine injection. Morphine treatment led to a 5–10% reduction in body weight that started on the third injection day and continued through each successive day in the experiment for both males (mixed-effects analysis: drug treatment F(1, 20) = 38.13, *p* < 0.0001, time F(4, 82) = 15.99, *p* < 0.0001, drug treatment × time interaction F(5, 98) = 12.90, *p* < 0.0001; Šídák post hoc test for multiple comparisons: Day 2: *p* = 0.11, Day 3: *p* < 0.001, Day 4: *p* < 0.01, Day 5: *p* < 0.001, Day 6: *p* < 0.0001; Fig. 4a) and females (mixed-effects analysis: drug treatment F(1, 21) = 26.37, *p* < 0.0001, time F(3, 59) = 3.88 *p* = 0.01, drug treatment × time interaction F(5, 99) = 11.09, *p* < 0.0001; Šídák post hoc test for multiple comparisons: Day 2: *p* = 0.07, Day 3: *p* < 0.01, Day 4: *p* < 0.001, Day 5: *p* = 0.02, Day 6: *p* = 0.04; Fig. 4b).

There are conflicting reports of sex differences in physical withdrawal signs in rodents. In some cases, males exhibit more severe and persistent withdrawal than females^27,36–38^. In other cases, females have more severe^39^ or more persistent withdrawal^26,40^. Here, both male and female mice exhibited physical characteristics associated with opioid dependence following the 5-day morphine injection procedure (2-way ANOVA: sex F(1, 32) = 0.27, *p* = 0.61, drug treatment F(1, 32 = 62.69, *p* < 0.0001, sex × drug treatment interaction F(1, 32) = 0.00, *p* = 0.99; Tukey post hoc test for multiple comparisons: male *p* < 0.0001, female *p* < 0.0001; Fig. 4c), indicating its suitability for investigating withdrawal-induced brain changes.

## Discussion

While there are numerous preclinical studies identifying sex differences in opioid-related behaviors^38,41–46^, sex differences in VTA GABA circuitry following opioids have not been examined. Consistent with previous literature, male mice showed reduced PPR and increased sIPSC frequency in the VTA during morphine withdrawal after a 5-day morphine injection procedure. On the other hand, morphine-treated female mice did not exhibit changes in PPR or sIPSCs. We also demonstrated that morphine treatment led to impairment of a form of inhibitory plasticity in males, whereas inhibitory plasticity remained intact in females and may proceed via a different synaptic mechanism.

The presynaptic increase in release probability observed during opioid withdrawal is mediated by adenylyl cyclase and cyclic adenosine monophosphate activity (cAMP) in males^17,47^. In the present study, the increased baseline GABA release in morphine-treated males likely occluded further HFS-induced potentiation of the synapses. This contrasts with a single morphine exposure, which blocks LTP_GABA_ without occlusion, whether morphine is applied to the slice or the animal is injected 2 h or 24 h prior to measurement^48^. In drug naïve animals, LTP_GABA_ proceeds through cGMP activity in the presynaptic GABA neurons^21^. Morphine, which inhibits cAMP activity^49^, presumably also acts on the cGMP pathway to disrupt LTP_GABA_ acutely, but the precise mechanism is unclear^50^. While LTP_GABA_ induction does not require cAMP, prior cAMP activation (for example bath application of forskolin) can occlude future electrically evoked LTP_GABA_^50^. Therefore, we infer that GABA release caused by cAMP activation during morphine withdrawal occluded further HFS-induced potentiation of GABA synapses in our experiments. Additional mechanisms contributing to GABA activity during opioid withdrawal have been discovered in addition to cAMP-mediated inhibitory plasticity. During withdrawal from chronic morphine, there is impairment of the K^+^-Cl^−^ co-transporter KCC2 in VTA GABA neurons, which disrupts Cl^−^ homeostasis and increases GABA neuron activity^51^. In summary, there exists a transition over the course of repeated morphine administration and withdrawal in which converging sources increase GABA activity sufficiently to induce cGMP mediated LTP_GABA_ in vivo and, thereby, occlude future potentiation. Our finding that LTP_GABA_ is occluded during opioid withdrawal fits with the theory of a hypodopaminergic state that is common to stress^52–54^ and withdrawal from multiple drugs of abuse including opioids^13^, nicotine^55^, and cocaine^56^.

LTP_GABA_ occurred in both saline and morphine-treated females. If morphine occluded LTP_GABA_ in males, LTP_GABA_ in morphine-treated females may have remained intact because GABA release probability was not significantly altered. Unexpectedly, females did not exhibit a decrease in PPR following HFS regardless of drug treatment, which indicates that there may be a different mechanism underlying synaptic potentiation of inhibitory inputs for males and females. A recent report showed that caudal stimulating electrode placement produced an atypical form of LTP in the VTA that was not occluded by forskolin and did not decrease PPR^57^. Male and female mice were used in the previous study, but data were not reported by sex. The insensitivity to adenylyl cyclase activation and postsynaptic mechanism bear resemblance to our finding that females exhibited LTP_GABA_ after morphine without a decreased PPR, even though we used a rostral electrode placement. LTP_GABA_ is also input-specific^22,58^, suggesting that sex differences in GABA afferents may translate to expression of typical versus atypical mechanisms of potentiation.

There are many possible explanations for the sex difference in GABA signaling during opioid withdrawal, including variation in morphine sensitivity or timing, contributions from upstream brain areas, cycling sex hormones, or most likely a combination of multiple factors. The 5-day, 10-mg/kg morphine dose used here caused physical withdrawal signs, and other groups have shown that it is sufficiently rewarding to produce place preference in females^59^, but it is possible that more severe dosing schedules cause changes in VTA GABA transmission that alter the presently observed sex differences. While not the focus of these experiments, gonadal hormones likely contribute to the VTA response to opioids and merit further study. Female sex hormones influence dopamine neuron activity^60,61^, which in turn controls the extracellular dopamine concentration in the striatum^60,62,63^ and conditioned place preference to other drugs of abuse^60^. Male sex hormones also regulate mesolimbic dopamine activity and extracellular dopamine concentration in the prefrontal cortex^64–66^. As previously mentioned, there are sex differences in brain areas upstream of the VTA, and the difference in GABA signaling that we observed could originate from a number of brain areas including the BNST and RMTg.

An importance of GABA signaling in the VTA is its role in regulating downstream dopaminergic activity in the striatum, which involves convergence of multiple GABAergic plasticity pathways^51,67,68^, glutamatergic plasticity^69^, neuromodulators including orexin^70^, and epigenetic factors^71^. Future studies should explore sex as a biological variable in relation to excitatory/inhibitory balance and dopamine firing rates to gain a complete understanding of the physiological consequences of opioid withdrawal. In turn, this information could be leveraged to fine-tune current therapeutics or identify novel, non-opioid treatments for OUD.

Inhibitory neurotransmitter function is increasingly implicated in sex differences of various psychiatric diseases. Transcriptional changes in GABA function are implicated as major contributors to sex differences found in addiction^72,73^, major-depressive disorder^74^, and post-traumatic stress disorder^75^. The interaction between these conditions is yet another underexplored line of research; women with OUD have higher rates of comorbid anxiety/depression disorders, while men have higher rates of comorbid non-opioid substance use^76^. OUD presents a complex range of symptoms, comorbidities, and treatment outcomes that are all subject to individual differences including sex, and our study provides evidence that the VTA is a potential source of behavioral sex difference in opioid withdrawal.

## Methods

### Animals and drug treatment

A total of 45 adult (9–16 weeks old) male and female C57BL/6 wildtype mice (Jackson Labs) were used. After arrival in the animal facility at 8 weeks old, mice were given at least 1 week to adjust to the new environment. Mice were housed 4–5 animals per cage in a temperature and humidity-controlled facility under a 12-h reverse light/dark cycle (lights off at 10:00 A.M.). Behavioral tests took place in the animals’ active phase. Cages contained corncob bedding, and standard rodent chow and water were available ad libitum in their home cage. All experiments were approved by the Institutional Animal Care and Use Committee (IACUC) at the University of Pennsylvania and carried out in compliance with guidelines and regulations specified by IACUC and Animal Research: Reporting of In Vivo Experiments (ARRIVE)^77^.

### Drug treatment

Mice received twice-daily systemic injections of morphine (10 mg/kg, 1 mg/ml, i.p.) for 5 days at 8:00 A.M. and 3:00 P.M. Morphine sulfate (Spectrum Chemical) was dissolved in sterile saline. Control animals received equivalent injections (10 ml/kg) of sterile saline.

### Spontaneous withdrawal test

18–24 h after the final morphine injection, mice were placed individually in a clean cage with standard bedding and an inverted second cage on top (total dimensions: 28 cm L × 18 cm W × 25 cm H). After a 5-min acclimation period, behavior was recorded for 20 min using an ELP infrared camera positioned 45 cm to the side. Videos were scored for signs of physical withdrawal, including jumps, wet dog shakes, teeth chattering, and abnormal posture (counted once per 5-min bin) by an observer blind to experimental group and sex. Total withdrawal scores were calculated by summing these withdrawal symptoms and percent decrease in weight on Day 6.

### Ex vivo slice electrophysiology

Electrophysiological recordings were performed as previously described^67,78,79^. Horizontal VTA slices (230 μm) were cut using a Leica VT1200S Vibratome while the brain was immersed in ice cold sucrose solution: 205.0 mM sucrose, 2.5 mM KCl, 21.4 mM NaHCO3, 1.2 mM NaH2PO4, 0.5 mM CaCl2, 7.5 mM MgCl2, and 11.1 mM dextrose. Brains were bubbled with 95% oxygen, 5% CO_2_ throughout slicing and recording. After cutting, slices were incubated for 40 min in 32 °C artificial cerebrospinal fluid (aCSF) buffer containing 120.0 mM NaCl, 3.3 mM KCl, 25.0 mM NaHCO3, 1.2 mM NaH2PO4, 2.0 mM CaCl2, 1.0 mM MgCl2, 10.0 mM dextrose, and 20.0 mM sucrose. Following incubation, slices were transferred to a recording chamber and perfused with 32 °C aCSF at a rate of 2–3 ml/min. WPI glass patch pipettes were pulled with a Narishige PC-10 pipette puller and had resistances of 1.5–3 MΩ. Pipettes were filled with intracellular solution containing (in mM): 135.0 KCl, 12.0 NaCl, 2.0 MgATP, 0.5 EGTA, 10.0 HEPES, and 0.3 TrisGTP (pH 7.2–7.3). Recordings took place in whole-cell mode under voltage clamp at − 60 mV unless noted otherwise.

Throughout the experiments, slices were bathed with 6,7-dinitroquinoxaline-2,3-dione (DNQX, Sigma, 10 μM) to block AMPA receptor glutamate activity and strychnine (Sigma, 1 μM) to block glycine activity. Cells were excluded if the resistance varied by more than 30%. Liquid junction potentials were corrected prior to the recordings. I_H_ was collected using voltage steps ranging from − 40 to − 110 in 10 mV increments. Input resistance was measured using 50 ms, 5 mV voltage steps. Spontaneous IPSCs were collected after at least 10 min of antagonist application in the bath. For experiments containing evoked IPSCs, a bipolar tungsten stimulating electrode was placed approximately 200 μm rostral to the recording electrode. Paired pulses were evoked using two 100 μs stimulations separated by 50 ms given at 0.1 Hz for 5 min. Baselines for LTP_GABA_ experiments were established by recording the IPSC amplitude (pA) in response to 100 μs, 0.1 Hz stimulation for 10 min with a minimum of 5 min of stable responses. To induce LTP_GABA_, the recording mode was moved to voltage follower (I = 0) to allow the membrane potential to vary during HFS, which consisted of two, 1-s, 100 Hz trains separated by 20 s, a procedure that reliably produces LTP_GABA_ in drug naïve mice^21,22^. After HFS, evoked IPSCs were measured for an additional 20 min. Recordings were collected using a Multiclamp 700B amplifier (Molecular Devices), filtered at 10 kHz, digitized at 20 kHz using pClamp 10.5 (Digidata 1550, Molecular Devices), and analyzed off-line using Clampfit 10.7.

Recordings took place in neurons in the lateral VTA (parabrachial pigmented nucleus), medial to the medial terminal nucleus of the accessory optical tract and bordering the substantia nigra pars compacta. In this subregion, neurons were selected based on slow pace-making firing rates (< 7 Hz), large soma size, and the presence of hyperpolarization activated current (I_H_). These characteristics highly correlate with dopamine (tyrosine hydroxylase+) neurons^80^ and replicate sampling criteria for comparison to previous studies^17,21,48^_._ Using this method, there remains a possibility that a small percentage of the neurons are non-dopaminergic. Furthermore, a percentage of VTA dopamine neurons do not exhibit these electrophysiological characteristics, though they are mostly confined to the medial VTA^81,82^. Medial and lateral subdivisions of the VTA project to distinct brain regions such that our recording location in the lateral VTA likely samples from neurons projecting to the nucleus accumbens (NAc) lateral shell. VTA dopamine neurons from more medial locations preferentially project to the medial prefrontal cortex (mPFC) and NAc medial shell^82^.

### Data analysis

Withdrawal, PPR, sIPSC, and LTP_GABA_ data were analyzed using 2-way analysis of variance (ANOVA) to test for main effects of sex or drug treatment and interactions. Significance in an ANOVA was followed by the Tukey HSD test for multiple comparisons when appropriate. Spontaneous IPSC frequency distributions were analyzed using Kolmogorov–Smirnov (KS) tests. LTP_GABA_ and PPR following HFS were analyzed using two-tailed paired t-tests of IPSC amplitudes during the 5-min period prior to HFS compared to the 5-min period 15–20 min after HFS. Mouse weight was analyzed for each sex using a repeated measures mixed effect analysis followed by Šídák multiple comparisons test. All datasets were normally distributed based on Shapiro–Wilks tests for normality and are presented as mean ± SEM with individual data points representing individual mice. In cases when multiple cells were recorded from the same animal, data were averaged to create a single value per animal. Statistical analyses were performed using GraphPad Prism software version 9.3.1 (San Diego, CA), and the significance level was set at alpha = 0.05.

## Data Availability

The datasets generated during and/or analyzed during the current study are available from the corresponding author on reasonable request.
